# Preparation and characterization of paclitaxel palmitate albumin nanoparticles with high loading efficacy: an in vitro and in vivo anti-tumor study in mouse models

**DOI:** 10.1080/10717544.2021.1921078

**Published:** 2021-06-10

**Authors:** Hang Chen, Sifan Huang, Heyi Wang, Xinmei Chen, Haiyan Zhang, Youfa Xu, Wei Fan, Yun Pan, Qiuyan Wen, Zhizhe Lin, Xuena Wang, Yongwei Gu, Baoyue Ding, Jianming Chen, Xin Wu

**Affiliations:** a Department of Pharmacy, Fujian University of Traditional Chinese Medicine, Fuzhou, China; b Department of Pharmacy, Inner Mongolia Medical University, Huhhot, China; c Shanghai Wei Er Biopharmaceutical Technology Co., Ltd, Shanghai, China; d Department of Pharmacy, Seventh People's Hospital of Shanghai University of Traditional Chinese Medicine, Shanghai, P.R. China; e School of Pharmacy, Jiaxing College, Jiaxing, China

**Keywords:** Paclitaxel palmitate, albumin nanoparticles, toxicity, tissue distribution, pharmacodynamics

## Abstract

**Background:**

Combination of the prodrug technique with an albumin nano drug-loaded system is a novel promising approach for cancer treatment. However, the long-lasting and far-reaching challenge for the treatment of cancers lies in how to construct the albumin nanometer drug delivery system with lead compounds and their derivatives.

**Methods:**

In this study, we reported the preparation of injectable albumin nanoparticles (NPs) with a high and quantitative drug loading system based on the Nab^TM^ technology of paclitaxel palmitate (PTX-PA).

**Results:**

Our experimental study on drug tissue distribution in vivo demonstrated that the paclitaxel palmitate albumin nanoparticles (Nab-PTX-PA) remained in the tumor for a longer time post-injection. Compared with saline and paclitaxel albumin nanoparticles (Abraxane^®^), intravenous injection of Nab-PTX-PA not only reduced the toxicity of the drug in normal organs, and increased the body weight of the animals but maintained sustained release of paclitaxel (PTX) in the tumor, thereby displaying an excellent antitumor activity. Blood routine analysis showed that Nab-PTX-PA had fewer adverse effects or less toxicity to the normal organs, and it inhibited tumor cell proliferation more effectively as compared with commercial paclitaxel albumin nanoparticles.

**Conclusions:**

This carrier strategy for small molecule drugs is based on naturally evolved interactions between long-chain fatty acids (LCFAs) and Human Serum Albumin (HSA), demonstrated here for PTX. Nab-PTX-PA shows higher antitumor efficacy in vivo in breast cancer models. On the whole, this novel injectable Nab-PTX-PA has great potential as an effective drug delivery system in the treatment of breast cancer.

## 1. Introduction

Surgery, radiotherapy and chemotherapy remain the mainstay of treatment for malignant tumors. Although gene therapy and monoclonal antibody therapy have good therapeutic efficacy, they adapt to limited patients due to the high cost **(**Hong et al., 2012**)**. Among various chemotherapeutic drugs, Paclitaxel (PTX) is the first-line drug for the treatment of breast cancer, but it may cause bone marrow suppression and hepatorenal toxicity, which reduces human tolerance to PTX. In the process of low effective dose use, drug resistance is easy to appear, leading to failure of treatment **(**Webster et al., 1993; Skwarczynski et al., 2006; Duggett et al., 2017**)**. Therefore, how to increase the dose of PTX via a PTX delivery system with low toxicity and high effectiveness is a hot topic and a challenging issue to be solved.

Due to the low-water solubility of PTX, polyoxyethylene castor oil (Cremophor EL) and ethanol are often used as solvents to prepare PTX injection. However, Cremophor EL is a surfactant that can easily cause severe allergic reactions, and, therefore, patients need to receive corticosteroids and antihistamines as pretreatment before medication. But even so, there are still more than 2% severe allergic reactions. In addition, the use of Cremophor EL makes PTX nonlinear elimination in the human body, which is not conducive to clinically safe use of drugs.

To avoid the use of Cremophor EL and reduce the toxic and adverse effects of the drug, PTX for injection (albumin binding) (paclitaxel albumin nanoparticles) was then marketed by the American Abraxis BioScience Company years ago. Studies have shown that paclitaxel albumin nanoparticles can alleviate a variety of adverse reactions and allergic reactions caused by Cremophor EL, and improve patient compliance and tolerance. As for poor solubility of paclitaxel itself, traditional paclitaxel preparations often exist, some matter that the low stability, high particle size, high polydispersity index (PDI) and the preparation process are difficult **(**Tai et al., 2016; Itatani et al., 2018**)**.

Our prior study found that PTX could be covalently combined with palmitic acid, forming a new compound known as Paclitaxel Palmitate (PTX-PA), which could significantly improve the liposolubility of PTX. After entering the body, PTX was released through esterase metabolism in tumor cells, where it exerted its anti-tumor effect. PTX-PA did not cause vascular stimulation and harm to the body during drug administration as compared with PTX alone. In addition, PTX-PA was more tolerable and could increase the dose of PTX by two fold. The experiment with PTX-PA metabolism in-vivo showed that PTX-PA not only increased the dose of PTX but also prolonged the released and circulation time of PTX.

Albumin can bind closely and reversibly with hydrophobic PTX through noncovalent bonds to realize the transport and release of the transported substances in vivo and has become a natural carrier of hydrophobic substances **(**Yin et al., 2015; Thadakapally et al., 2016; Tai et al., 2017**)**. In the present study, we used albumin to prepare Nab-PTX-PA via Nab^TM^ technique. We found that the procedure of preparing Paclitaxel Palmitate Albumin Nanoparticles (Nab-PTX-PA) was relatively simple and stable, and the particle size and the polymer dispersity index (PDI) were significantly lower than those of paclitaxel albumin nanoparticles. Subsequently, we further evaluated the particle size, Zeta potential, in-vitro release, physical stability and loading capacity of the albumin nanoparticles (NPs). Cytotoxicity and cellular uptake of Nab-PTX-PA in 4T1 breast cancer cells in Institute of Cancer Research (ICR) mice were evaluated. In addition, tissue distribution, the anti-tumor effect and safety of Nab-PTX-PA were also evaluated.

## 2. Material and methods

### 2.1. Materials

Materials used in this study included PTX (purity > 98%; Jiangsu Yew Biotech Co., Ltd, Wuxi, China), PTX-PA (purity > 97%) and Nab-PTX-PA (our laboratory), 20% Human Serum Albumin (HSA (MW ≈ 66 KD, (10 g/50 mL)), Shanghai Lloyd Blood products Co., Ltd, Shanghai, China), paclitaxel albumin nanoparticles (Fresenius Kabi USA), heparin sodium (Guoyao Group Chemical Reagent Co., Ltd, Shanghai, China), and Coumarin 6 (Aladdin Reagent Platform), CCK-8 and DMSO (Sigma-Aldrich, Shanghai). All other solvents were of chromatographic grade and other chemicals were of analytical grade. Twenty-four female ICR mice weighing 18 ± 2 g (Second Military Medical University, Shanghai, China) were housed at 25 ± 2 °C with relative humidity at 47.5 ± 2.5%. All animals in this study were from the Animal Center of the second military Medical University, and the animal experiments were completed at the Second Military Medical University. One thing we can guarantee is that all animal procedures were performed in accordance with the Ethical Guidelines for Investigations in Laboratory Animals issued by the Second Military Medical University. Care and handling of the animals were in strict compliance with the “Guide for the Care and Use of Laboratory Animals” of China.

### 2.2. Preparation of the albumin NPs

The preparation method of Nab-PTX-PA for injection was as follows: Simply, PTX-PA (447 mg) was dissolved in chloroform (1.97 mL) and anhydrous ethanol (0.3 mL) to acquire the organic phase. HSA (12.5 mL) was mixed with appropriate amount of distilled water (volume to 50 mL) to obtain the aqueous phase. Then, when its temperature dropped to 0 °C, the organic phase was added to the shearing machine by continuous stirring and dispersing the water phase, and the colostrum was obtained by shearing for 1 min at 10000 r/min stirring, which was homogenized and emulsified at 15000 psi for four times by using a high pressure homogenizer to obtain Nab-PTX-PA. The organic solvent was removed by ultrafiltration, and then separately packaged, freeze-dried and cap-sealed to obtain its lyophilized products.

### 2.3. Characterization of the albumin NPs

The particle size and PDI of the formulation were measured with a Zetasizer laser particle size analyzer (Malven, UK). In addition, the morphology of the preparation was characterized by transmission electron microscopy (TEM) via negative staining. The sample preparation for TEM photography was as follows: Nab-PTX-PA solution was dripped on the surface, let settle into the carbon-sprayed copper mesh, negatively stained with 2% phosphotungstic acid for 3 min, and dried naturally at high resolution. The morphology of Nab-PTX-PA was observed by TEM.

### 2.4. Stability, drug loading capacity(DL%) and encapsulation efficiency(EE%) study

The physical stability of PTX-PA solution was examined in a sealed and dark condition at 4 °C. Briefly, at different intervals (0, 4, 12 and 24 weeks post-preparation), samples from solution were regularly collected at the designated time points to evaluate the physical stability.

For the determination of the Encapsulation Efficiency (EE%) and Drug Loading capacity (DL%) of nanoparticle, ultrafiltration centrifugation is mostly used. One millilitre Nab-PTX-PA in 30 kD ultrafiltration centrifuge tube was cebtrifuged at 10,000 r/min for 15 min. The same concentration of 1 mL Nab-PTX-PA, with methanol was taken in a 10 mL capacity bottle. The resultant free drug content in filtrate and total drug content in formulation were analyzed by HPLC for the drugs. The content of PTX-PA in NPs formulation was calculated by subtracting the amount of free PTX-PA from the total PTX-PA. EE (%) was measured based on Equation (1), and DL (%) was measured based on Equation (2).

(1) $$\text{EE}\%=\left({\text{C}}_{0}-{\text{C}}_{\text{1}}\right)/{\text{ C}}_{0}\times \text{1}00\%$$

(2) $$\text{DL}\left(\%\right)={\text{W}}_{\text{E}}/\left({\text{W}}_{\text{T}}+{\text{W}}_{\text{L}}\right)\times \text{1}00\%$$

C_1_: Free drug content in filtrate. C_0_: Total drug content in formulationWhere W_E_, W_T_, W_L_ were the weight of PTX-PA entrapped in the freeze-dried product (1 mL, Nab-PTX-PA), the weight of total PTX-PA, and the weight of albumin added in the system (the weight of freeze-dried product (1 mL, Nab-PTX-PA), respectively.

### 2.5. Drug release experiments

The release kinetics of paclitaxel palmitate in albumin NPs was quantitatively detected by drug release experiments. 1 mL Nab-PTX-PA was mixed with 200 mL release phosphate buffer solution (10% ethanol, pH5.5). Samples collected at various time points were dissolved by paddle stirring at a rotation speed of 100 rpm, and the concentrations of the entrapped drugs were determined after removing the released drug by filter membrane filtration. The sample solution (100 μL) was absorbed in a 1.5 mL centrifuge tube, and the protein was precipitated with 300 µL acetonitrile. After vortex-mixing for 1 min, the sample was centrifuged at 13,000 rpm for 10 min. Finally the supernatant (20 μL) was analyzed by HPLC.

### 2.6. *In vitro* plasma conversion

First, the blood was collected from the orbital plexus of rats with capillary tube to put into heparin-containing tubes, and centrifuged at 4,000 rpm for 10 min to obtain the upper plasma (blank plasma). Then freeze-dried powder of Nab-PTX-PA (1.093 mg) was weighed in the cylinder, dissolved with 2 mL SD rat blank plasma (PTX-PA concentration 100 μg·mL^−1^), and centrifuged at low speed of 100 rpm min^–1^, in a shaker at 37 °C with shock incubation. Plasma samples (0.1 mL) were collected from the cylinder at specific time intervals (0, 1, 3, 5, 7, 9, 12, 24 and 30 h) . Subsequently, 100 μL plasma was spiked with 10 μL of internal standard (100 μg/mL of Docetaxel (DTX)) , and then vortex-mixed with 1 mL methyl tert-butyl ether for 2 min. The organic layer was gathered after being centrifuged at 10000 rpm for 15 min and evaporated under a steam of nitrogen. The residue was redissolved in 100 μL of methanol, then vortexed for 5 min and centrifuged at 10000 rpm for 10 min, finally supernatant (20 μL) of sample was analyzed by HPLC to determine the concentration of PTX.

### 2.7. Maintenance of cell lines

Mouse breast cancer 4T1 cells were kindly provided by Stem Cell Bank of the Chinese Academy of Sciences (Shanghai, China) and cultured in DMEM medium, and maintained in the 5% CO_2_ atmosphere in a humidified incubator at 37 °C **(**Karimi et al., 2020**)**.

### 2.8. Cytotoxicity assay

Cytotoxicity of different formulations was measured by CCK-8. 4T1 cells were seeded in 96 well plates at a density of 5 × 10^3^ cells per well, pre-cultured in a 37 °C and 5% CO_2_ incubator for 24 h, treated with a series of Nab-PTX-PA and paclitaxel albumin nanoparticles working solutions, and incubated at 37 °C for additional 48 h. After adding 10 μL CCK-8 in each well, incubation was continued for 2 h. Absorbance was measured at 570 nm and the mean value was used for analysis. The cell survival rate was calculated using the following equation (Shahani et al., 2010; Yi et al., 2015; Salehiabar et al., 2018):

Cell survival rate (%)=[(As−Ab)/(Ac−Ab)]×100%

Label: As: experimental hole (culture medium containing cells, CCK-8 reagents, drugs). Ab: blank pore (medium without cells and drugs, CCK-8 reagent. Ac: control hole (cell-only medium and CCK-8 reagent).

### 2.9. *In vitro* cell uptake

Albumin NP uptake assay was performed by flow cytometry. 4T1 cells were plated with 100 μL Coumarin 6, Nab-PTX-PA and paclitaxel albumin nanoparticles fluorescent NPs and incubated for 4 h **(**Karimi et al., 2013**)**. The culture medium was centrifuged at 1,000 r/min for 5 min. The supernatant was removed by 1 mL PBS centrifugation for two times and then mixed with 500 μL PBS. The mean fluorescence intensity of 10,000 cells was calculated by using a 527 nm passband filter FL1-H to detect green fluorescence at 490 nm by flow cytometry, and changes in size of the PTX-PA fluorescent albumin NPs and paclitaxel albumin nanoparticles fluorescent NPs were measured using a Malvern particle size analyzer **(**Xiong et al., 2017; Dou et al., 2019**)**.

### 2.10. Tissue distribution assay

Thirty-six ICR mice were randomly divided into two groups and injected with paclitaxel albumin nanoparticles 20 mg/kg and Nab-PTX-PA 25.58 mg/kg via the tail vein **(**Chung et al., 2020**)**. Mice were sacrificed at 0.5, 1, 4, 8, 24 and 48 h post injection. Blood samples and tissues including the heart, liver, spleen, lung, kidney and tumor were collected and weighed at 1, 4 and 24 h **(**Pandya et al., 2019; Li et al., 2019**)**. Tissue samples were prepared at a 1:3 (w/w) ratio with normal saline (NS) and ground in a high-speed homogenizer into homogenates. One hundred microlitres of tissue homogenate was transferred into a 1.5 mL centrifuge tube, and protein was precipitated with 300 μL acetonitrile containing internal standard liquid **(**Gardner et al., 2008; Verco et al., 2019**).** The supernatant was vortically mixed for 1 min and centrifuged at 13000 r/min for 10 min, from which 150 μL supernatant was mixed with 300 μL 30% acetonitrile and prepared into a 5 μL sample injection, analyzed by HPLC-MS to determine the concentration of PTX-PA and PTX.**(**Kaddoumi et al., 2017).

### 2.11. *In vivo* antitumor effects

4T1 cells of logarithmic growth were diluted by addition of an appropriate amount PBS and injected into the right axilla of the ICR mice at a 0.2 mL concentration of about 1 × 10^7^/mL of 4T1 cell suspension. ICR mice were inoculated with ∼10^7^ 4T1 cells on the right flank, and treatments began when average animal tumor burden was between 50 and 150 mm^3^.

Twenty-four tumor-bearing ICR mice were equally randomized into four groups receiving 0.2 mL NS, paclitaxel albumin nanoparticles (20 mg/kg), Nab-PTX-PA (25.58 mg/kg) and double-dose Nab-PTX-PA (51.16 mg/kg) through the tail vein once every other day, totaling four times. Each mouse tumor volume was calculated according to (a^2^×b)/2 formula(the short diameter (a) and long diameter (b)) . The relative tumor volume was calculated according to the RTV = V_t_/V_0_ **(**Kinoshita et al., 2017), where the tumor volume was V_0_ when randomly grouped, and the tumor volume at each measurement was V_t_, 24 h after the mice were weighed and sacrificed. The tumor inhibition rate and relative tumor proliferation rate were calculated according to the following formula:

$$\text{relative}\;\text{tumor}\text{​}\text{​}\;\text{proliferation}\;\;\text{rate}\;\;=\;\frac{\text{RTV}\;\text{of}\;\text{administation}\;\text{group}}{\text{RTV}\;\text{of}\;\text{control}\;\text{group}}\text{​}\text{​}\;\times 100\%$$

### 2.12. Safety evaluation

#### 2.12.1. Blood routine examination

In the pharmacodynamics experiment, after 24 h ICR mice were last intravenously (IV) administered, 0.5 mL whole blood was drawn from the orbit of the ICR tumor-bearing mice with a trace capillary needle and treated with the anticoagulant for blood routine counting, including white blood cell (WBC), hemoglobin (HGB), red blood cell (RBC), neutrophil (Neut) and platelet (PLT) **(**Okamoto et al., 2019**)**.

#### 2.12.2. Blood biochemical test

After the last 24 h pharmacodynamics, 0.5 mL anticoagulant-treated whole blood from the ICR tumor-bearing mice was collected in a dry tube. Albumin (ALB), total bilirubin (TBIL), alanine aminotransferase (ALT), alanine aminotransferase (AST), creatinine (CRE), urea nitrogen (UN) and creatine kinase (CK) were the main indexes for blood biochemical detection.

### 2.13. The method of analysis sample using HPLC or HPLC-MS/MS

Using Agilent 1260II HPLC system for high performance liquid chromatography (HPLC) analysis(Agilent, USA). Samples were separated using an Agilent EclipsePlus C18 column (250 × 4.6 mm, 5 μm). The mobile phase was a mixture of methanol & water (95:5, v/v), and the flow rate was 1.0 mL/min. Absorbance was monitored at 227 nm.

Using Agilent Eclipse plus C18 column (4.6 mm × 250 mm, 5 μm), gradient elution was performed at a UV detection wavelength of 227 nm. The elution program was water(A): acetonitrile(B), 0-14 min: 68%A-32%B; 1 4∼27 min: 100%A-0%B; 2 7 ～28 min: 68%A-32%B, 2 9 ∼ 33 min and the flow rate of 1 mL/min, inject 20 μL of the plasma sample to be tested.

MS conditions are shown in the table below: 

**Table:** 

Mass Spectrometer Conditions				
Ionization Mode	ESI (+)			
Mass Spectrometer Conditions:				
Compound ID	Q1	Q3	fragmentor	CE
PTX-PA	1114.5	546.3	240	32
	1114.5	308.1	240	60
PTX	876.3	308.1	195	28
	876.3	591.2	195	24
IS:Dic	296	250.1	90	8

### 2.14. Statistical analysis

Data were statistically analyzed using GraphPad Prism (version 6, San Diego, USA) and SPSS22 software. All studies were repeated three times and all measurements were carried out in triplicate. Results are reported as the mean ± standard deviation (SD). C was tested by factor analysis of variance. Differences between experimental groups were considered significant when the *p*-Value was less than .05.

## 3. Results

### 3.1. Physicochemical characterization of albumin NPs of PTX-PA

**Figure 1.**
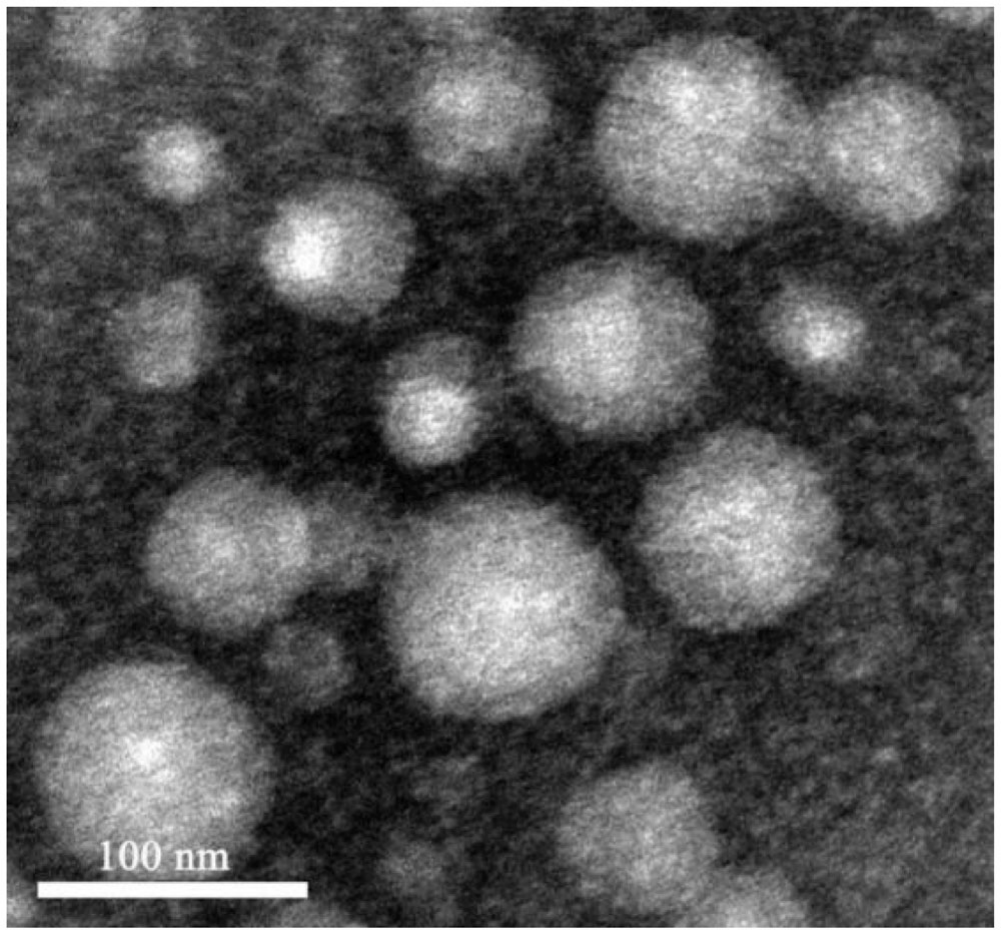
TEM micrographs of Nab-PTX-PA.

**Figure 2.**
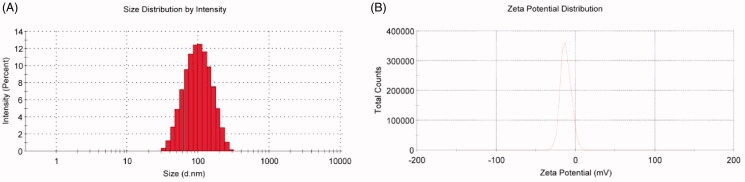
Particle size distribution and zeta potential of Nab-PTX-PA.

According to TEM, the appearance of Nab-PTX-PA was spherical and evenly distributed (Figure 1). Its particle size was 87.63 ± 1.15 nm, with a single peak of 0.185 ± 0.009 and a Zeta potential of 11.7 ± 0.61 mV. The typical particle size distribution and Zeta potential are shown in Figure 2.

### 3.2. Stability test, DL% and EE% study

**Figure 3.**
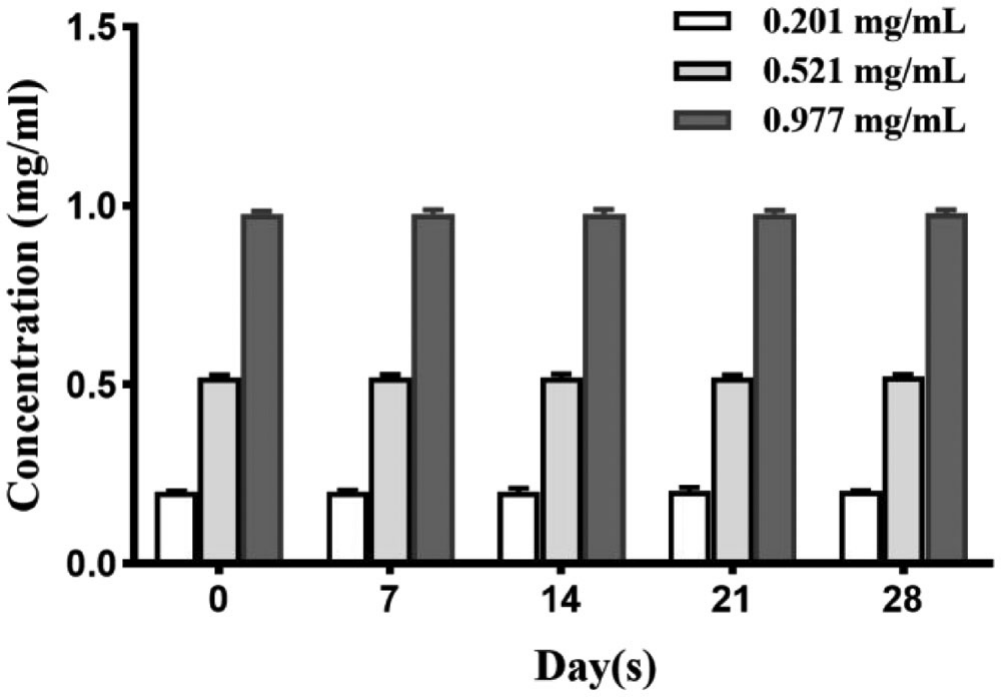
Stability of three PTX-PA solutions of different concentrations.

The stability of PTX palmitate solution under 4 °C conditions is shown in Figure 3. The results showed that the content of three different concentration of PTX-PA solution at 4 °C remained basically unchanged with good stability in one month. As Table 1 shows, the average encapsulation efficiency of three preparations by ultrafiltration centrifugation was 97.71 ± 0.49. The results of this method were stable and the error was small to meet the experimental requirements. Weighing freeze-dried samples (1 mL Nab-PTX-PA), and after redissolution put into 30 kD ultrafiltration centrifuge tube and centrifugated at 10000 r/min for 15 min. The resultant free drug content in filtrate and total drug content in formulation were analyzed by HPLC for the drugs. The content of PTX-PA entrapped in NPs formulation was calculated by subtracting the amount of free PTX-PA from the total PTX-PA. Then actual weight of PTX-PA loaded in reeze-dried samples divided by the total weight of freeze-dried samples (1 mL Nab-PTX-PA). The average drug loading capacity (%) of three preparations was 18.30 ± 0.31 in Table 2.

**Table 1.** The encapsulation rate of Nab-PTX-PA (*n* = 3).

Time	C_0_ (μg/mL)	C_1_(%)	EE（%）	Mean ± SD
1	6.49	0.13	98.17	97.71 ± 0.49
2	7.21	0.15	97.90	
3	7.31	0.21	97.01	

**Table 2.** The drug loading capacity of Nab-PTX-PA (Freeze-dried products) (*n* = 3).

Freeze-dried samples (1mL Nab-PTX-PA)	W_E_ (mg)	W_total_ (mg)	DL (%)	Mean ± SD
1	8.76	48.24	18.16	18.30 ± 0.31
2	8.75	48.58	18.01	
3	8.68	46.35	18.73	

### 3.3. Drug release experiments

Using the method of dissolution apparatus, the mixed solution of ethanol and PBS, after adjusting the pH value to 5.5, was used as the dissolving medium. Moreover, we did PBS (pH 5.5) and PBS (pH5.5) to add different amounts of ethanol as release medium in-vitro. The results showed that the drug release was slow in the pH5.5 alone. And the cumulative release of the platform period is only 40%. The addition of appropriate amount of ethanol can obviously accelerate the release of drugs, and can increase the cumulative release of drugs to more than 90%.

**Figure 4.**
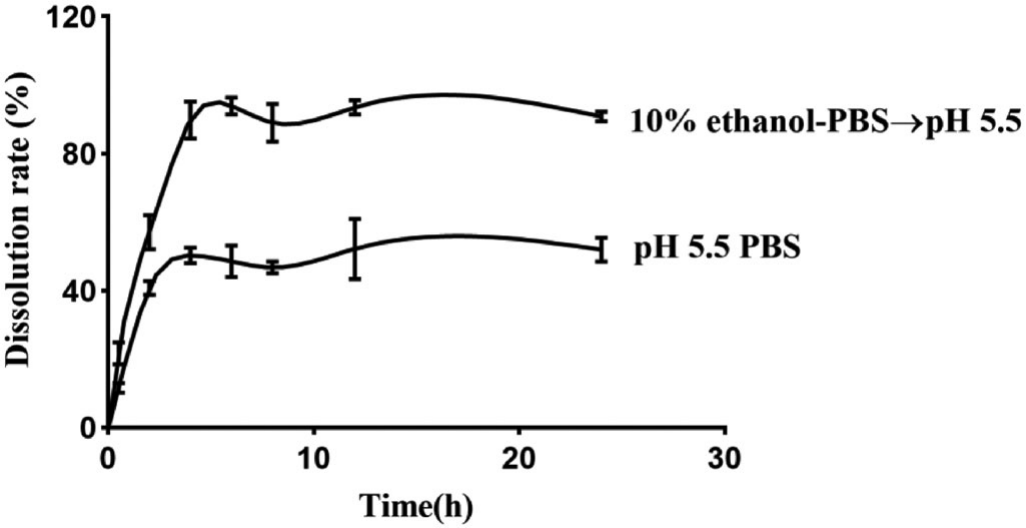
*In-vitro* release of Nab-PTX-PA in different media.

By analogy with the release of Nab-PTX-PA in different concentrations of ethanol, the release rate of two media (PBS (pH 5.5) and the mixed solution (pH5.5) of 10% ethanol and PBS) was found to be relatively superior. In the mixed solution (pH5.5) of 20% ethanol and PBS, the release rate of Nab-PTX-PA reached over 90% at 2 h but the release rate of the drug was too fast. The release rate of PBS (pH5.5) at 4 h was about 50%, showing relative stability in Figure 4. In the mixed solution (pH5.5) of 20% ethanol and PBS, the release rate of Nab-PTX-PA reached 90% and remained stable for 6 h. In both media, the release rate of Nab-PTX-PA was satisfactory and there was no obvious release acceleration.

### 3.4. *In vitro* plasma transformation

**Figure 5.**
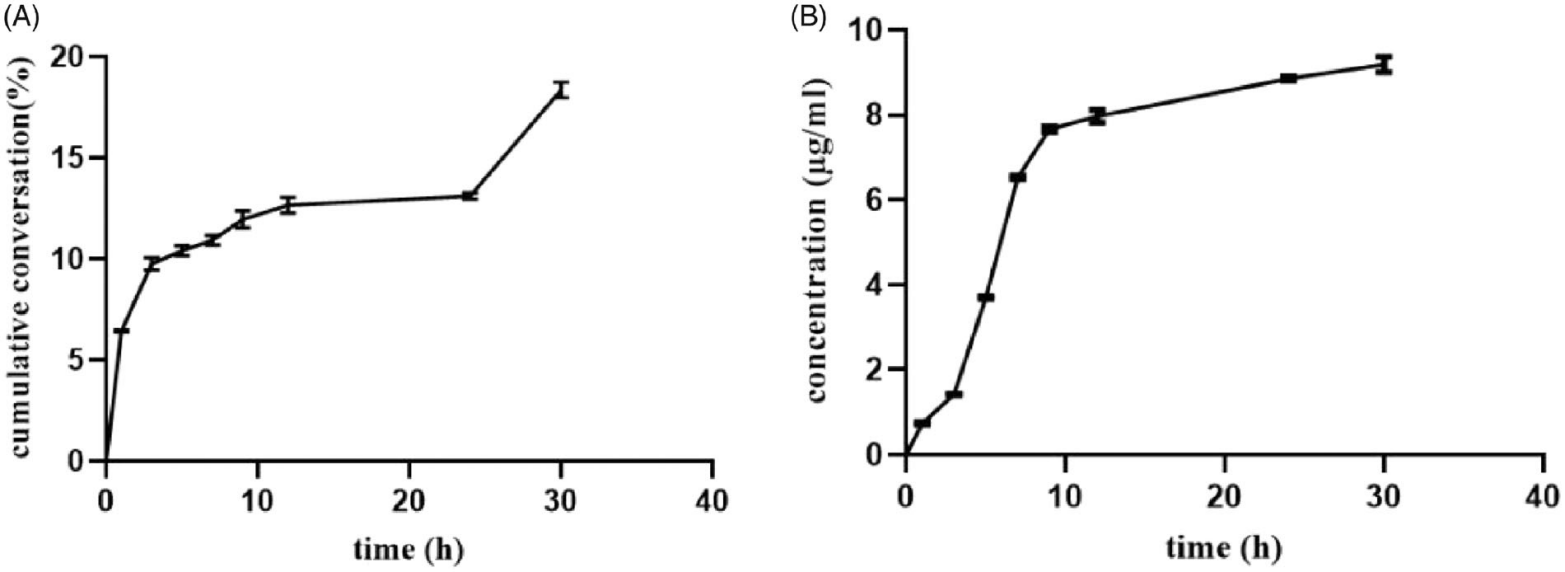
The cumulative conversion curve of Nab-PTX-PA in rat plasma and the converted PTX concentration (*n* = 3). (A) The cumulative conversion curve of Nab-PTX-PA in rat plasma, (B) the concentration of PTX.

**Figure 5** shows the cumulative conversion rate in rat plasma in Nab-PTX-PA at 30 h and the concentration of PTX. The results showed that about 18.35% Nab-PTX-PA was released from 0 h to 30 h, indicating that the active drug PTX could be released slowly in rat plasma, which laid the foundation for subsequent animal experiments in vivo.

### 3.5. Cytotoxicity assay

**Figure 6.**
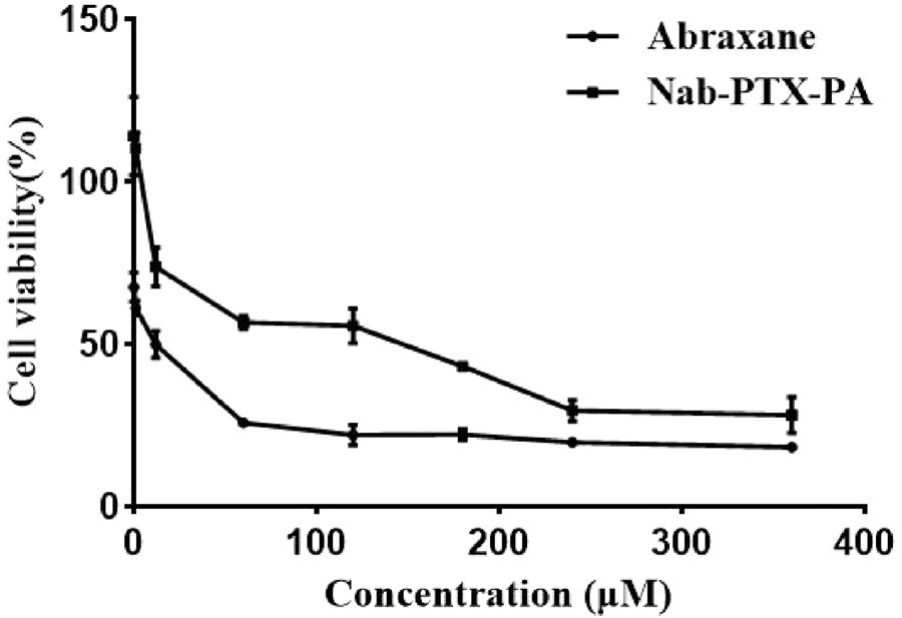
The 4T1 cell survival rate of Abraxane and Nab-PTX-PA at different concentrations (*n* = 3).

The cytotoxicity of Nab-PTX-PA and Abraxane was investigated by CCK-8 assays with 4T1 cells. As shown in Figure 6 and Table 2, the survival rate of 4T1 cells in different concentrations of Nab-PTX-PA was higher than that in paclitaxel albumin nanoparticles. Table 3 shows that the IC_50_ of Nab-PTX-PA was about 30 times that of paclitaxel albumin nanoparticles. In addition, paclitaxel albumin nanoparticles and Nab-PTX-PA both had concentration-dependent toxicity, and the toxicity of Nab-PTX-PA was lower than that of paclitaxel albumin nanoparticles at the same concentration (Figure 6).

**Table 3.** IC_50_ of Abraxane and Nab-PTX-PA.

Cell line	IC_50_ (μM)	
	Abraxane^®^	Nab-PTX-PA
4T1	3.498	103.7

### 3.6. Cell uptake

The preparation method of PTX-PA Albumin nanoparticles modified by Coumarin 6 (Cou-6-PTX-PA-NPs) for injection was as follows: Simply, 10 mg coumarin-6 was weighed and added into oil phase of “2.2” projects. Then the rest of the operation and follow-up steps were the same as under “2.2”, that was, Cou-6-PTX-PA-NPs. Finally centrifuged at 4500 rpm·min^−1^ for 15 min to remove the unwrapped coumarin-6. In addition, the fluorescent albumin nanoparticles of paclitaxel (PTX-NPs) were prepared according to the prescription of Abraxane.

**Figure 7.**
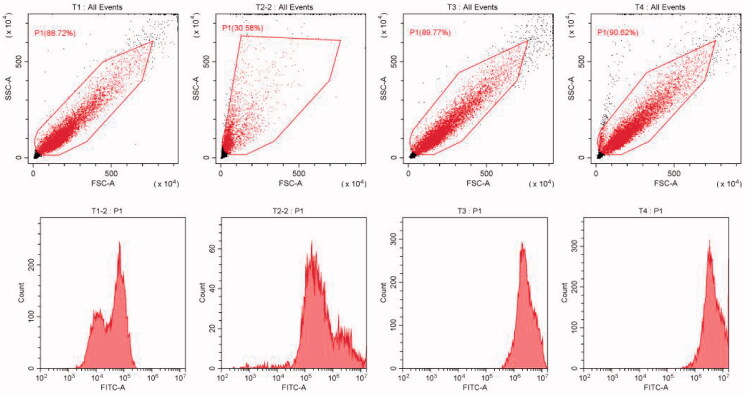
Cell flow chart of each group. (A) blank cell group, (B) free coumarin group, (C) Nab-PTX-PA fluorescence group, (C) Abraxane^®^ fluorescence group.

The cell uptake experiment showed in Figure 7 that the fluorescence intensity of Nab-PTX-PA and paclitaxel albumin nanoparticles cells was higher than that in Coumarin 6 group, demonstrating that both Nab-PTX-PA and paclitaxel albumin nanoparticles could improve 4T1 cell uptake. The PTX-PA uptake 4 h after Nab-PTX-PA was lower than that of paclitaxel albumin nanoparticles (Table 4), indicating that the Nab-PTX-PA entry process was slow and long-lasting as compared with paclitaxel albumin nanoparticles.

**Table 4.** The mean fluorescence value of each group.

Test tube name	Number of particles	Total (%)	Mean FITC-A
Blank cell Group	10000	100.00	30629.6
Coumarin 6 Group	10000	100.00	174358.4
Nab-PTX-PA Fluorescence Group	10000	100.00	2187230.5
Abraxane® Fluorescence Group	10000	100.00	3448224.3

### 3.7. Biodistribution

**Figure 8.**
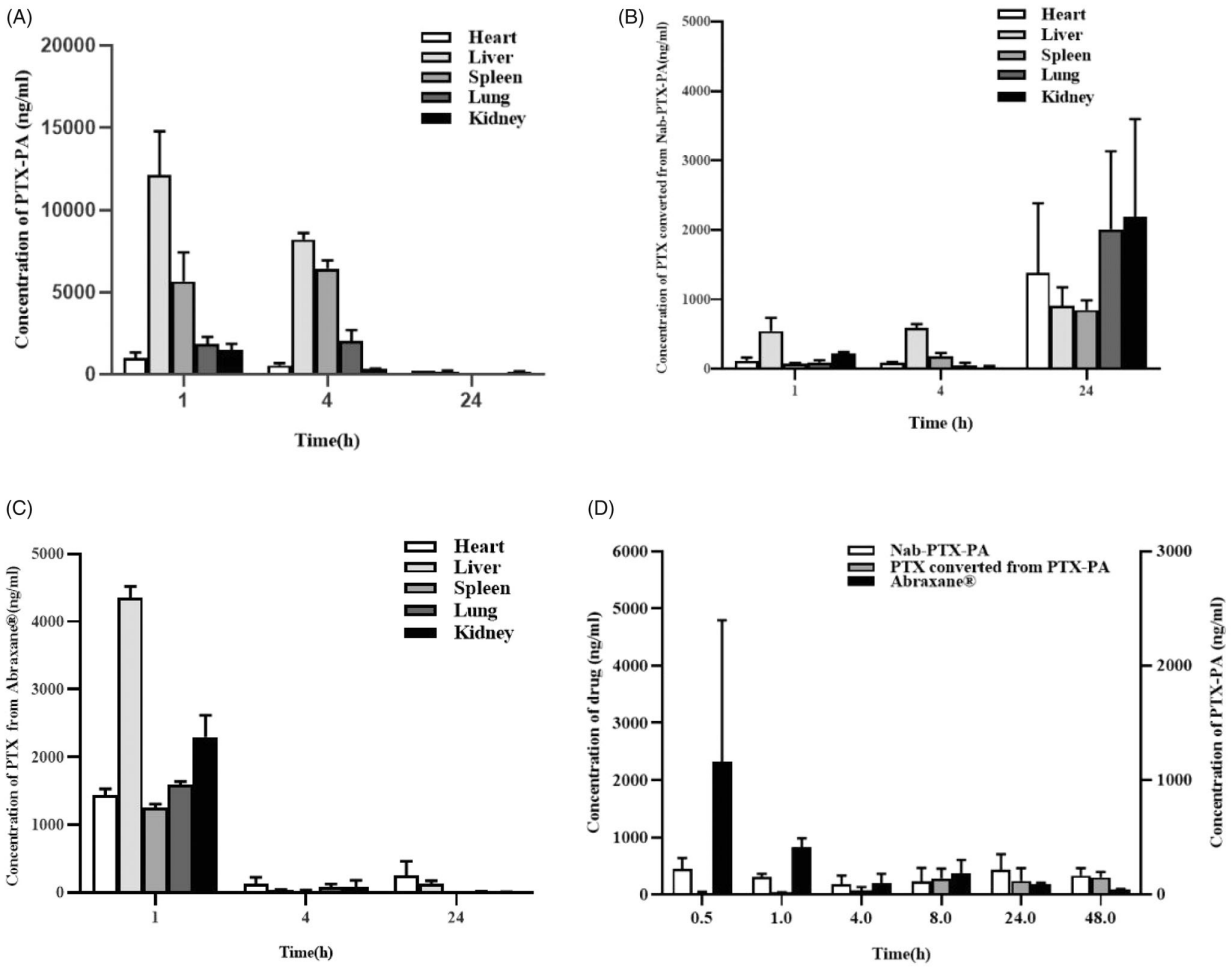
Concentrations of Abraxane and Nab-PTX-PA in tissues after intravenous injection in 4T1 tumor-bearing ICR mice. (A) Concentration of Abraxane, (B) Concentration of PTX-PA, (C) Concentration of PTX converted from PTX-PA, (D) Concentration of PTX in tumor.

The drug concentration-time distribution in the tumor-bearing mice is shown in Figure 8. the PTX of paclitaxel albumin nanoparticles was almost stored in the liver and kidney, and the concentration of it for which in the tumor site was relatively little, and the drug concentration gradually decreased with time. Figure 8(A) shows the concentration change of prodrug (PTX-PA) in each tissue after caudal vein injection, released from Nab-PTX-PA. The result showed that PTX-PA was mainly distributed in the liver, spleen and kidney. Figure 8(D) shows a slow increase in PTX-PA at the tumor site. This might be due to the tumor enhanced permeability and retention (EPR) effect, which caused slow entry of Nab-PTX-PA into the tumor. Figure 8 (B,C) shows drug distribution of PTX metabolized in the heart, liver, spleen, lung and kidney of the tumor-bearing mice. The result showed that the concentration of metabolic PTX distributed across all tissues less than paclitaxel albumin nanoparticles, as well as the metabolic PTX of Nab-PTX-PA less than paclitaxel albumin nanoparticles about which the toxicity for tissues. Figure 8 (D) shows the intuitive comparison of distribution of paclitaxel albumin nanoparticles and PTX-PA metabolized PTX in the tumor site. The result showed that the concentration of PTX in paclitaxel albumin nanoparticles decreased gradually with time, The PTX concentrations of metabolized PTX-PA increased over time, proving that PTX-PA could release active drug PTX slowly at the tumor site and stay in the tumor for a long time to play an anti-tumor role.

### 3.8. Antitumor study

**Figure 9.**
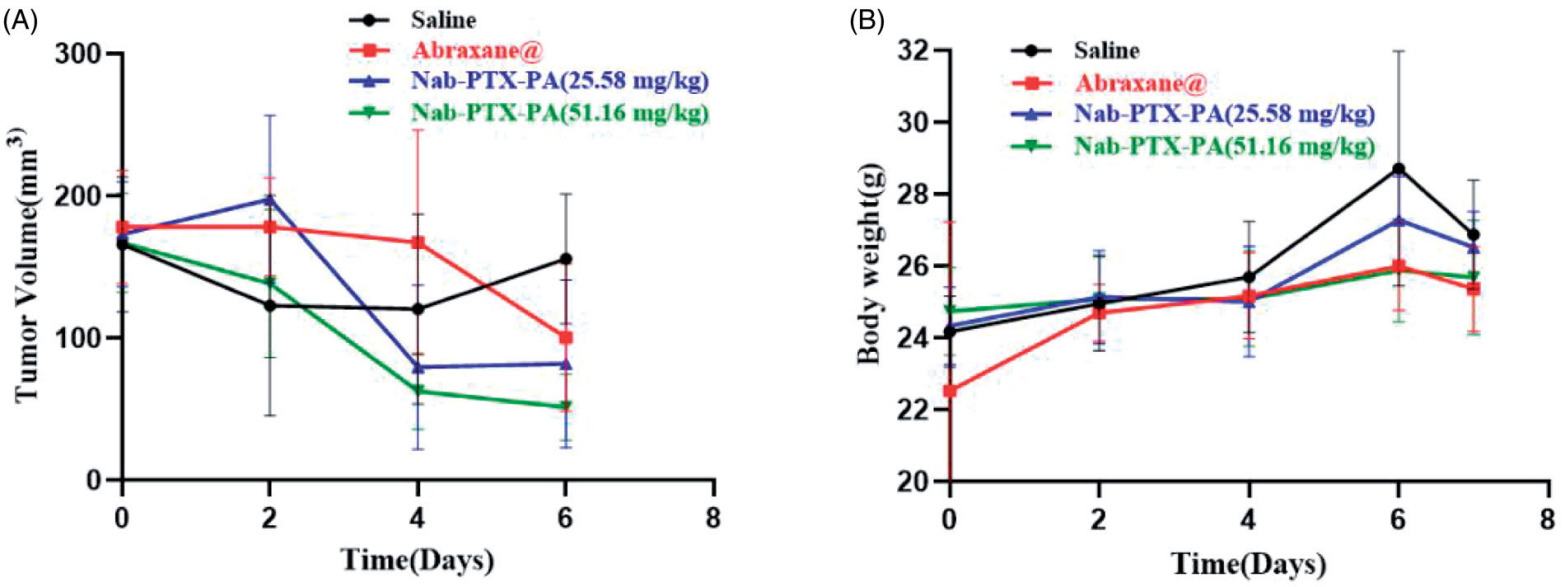
Graphs indicating tumor volume and weight changes in Abraxane, Nab-PTX-PA (25.58 mg/kg) and Nab-PTX-PA (51.16 mg/kg) mouse 4T1 cell models. (A) Tumor Volume Change Graph, (B) Weight Change Line Chart.

**Figure 10.**
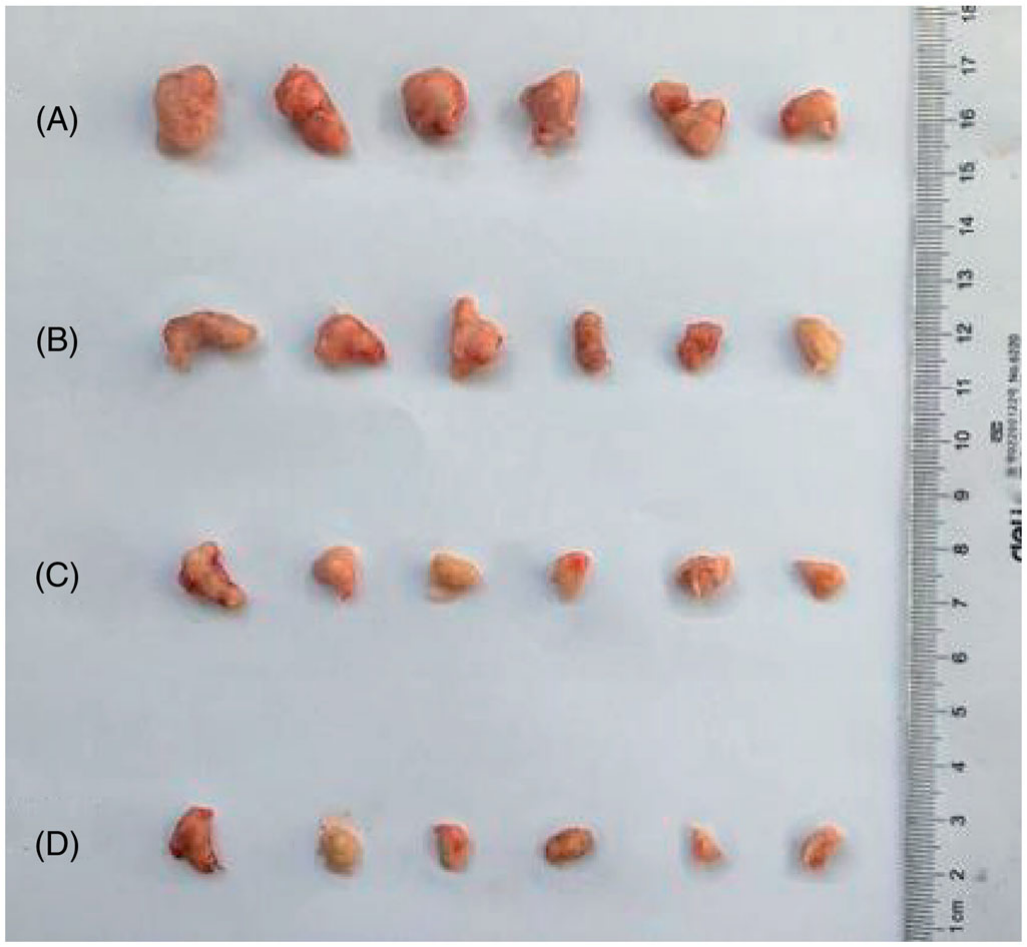
Antitumor effect diagram. (A) Normal saline group, (B) Abraxane group, (C) Nab-PTX-PA (25.58 mg/kg) group, (D) Nab-PTX-PA (51.16 mg/kg) group.

As shown in Table 5, paclitaxel albumin nanoparticles (20 mg/kg), Nab-PTX-PA (25.58 mg/kg) and Nab-PTX-PA (51.16 mg/kg) had different anti-tumor effects. The tumor inhibition rate of 25.58 mg/kg and 51.16 mg/kg Nab-PTX-PA was higher than that of paclitaxel albumin nanoparticles (20 mg/kg), with the tumor inhibition rate of 51.16 mg/kg Nab-PTX-PA being more significant. Figure 9 (A) shows that the tumor volume in the two Nab-PTX-PA groups decreased with time, and this decreasing trend was more significant in 51.16 mg/kg Nab-PTX-PA group. Figure 9 (B) shows the change in body weight of the tumor-bearing mice during drug administration. The results showed that the mouse weight in 25.58 mg/kg and 51.16 mg/kg Nab-PTX-PA groups were slightly elevated or remained basically unchanged, indicating that Nab-PTX-PA had fewer adverse effects as compared with the group of paclitaxel albumin nanoparticles (20 mg/kg). Figure 10 also shows that, Compared with the saline group, the mouse weight in 25.58 mg/kg and 51.16 mg/kg Nab-PTX-PA groups showed obvious antitumor effect.

**Table 5.** Antitumor effect of Nab-PTX-PA in the mouse breast cancer 4T1 model in terms of tumor weight ($\bar{x}\pm \text{s},$ *n* = 6).

Group	Animal	Tumor weight	Tumor suppressor	Relative tumor proliferation rate
	Start/End (number)	(g)	(%)	(%)
Saline	6/6	0.42 ± 0.23	—	—
Abraxane	6/6	0.22 ± 0.15*	47.62	64.08
Nab-PTX-PA(25.58 mg/kg)	6/6	0.20 ± 0.12*	52.38	50.61
Nab-PTX-PA(51.16 mg/kg)	6/6	0.13 ± 0.05*	69.05^&^	32.83^&^

Comparison with negative control (saline): *P<0.05

Comparison with Abraxane® group: &P<0.05

### 3.9. Blood routine examination

**Figure 11.**
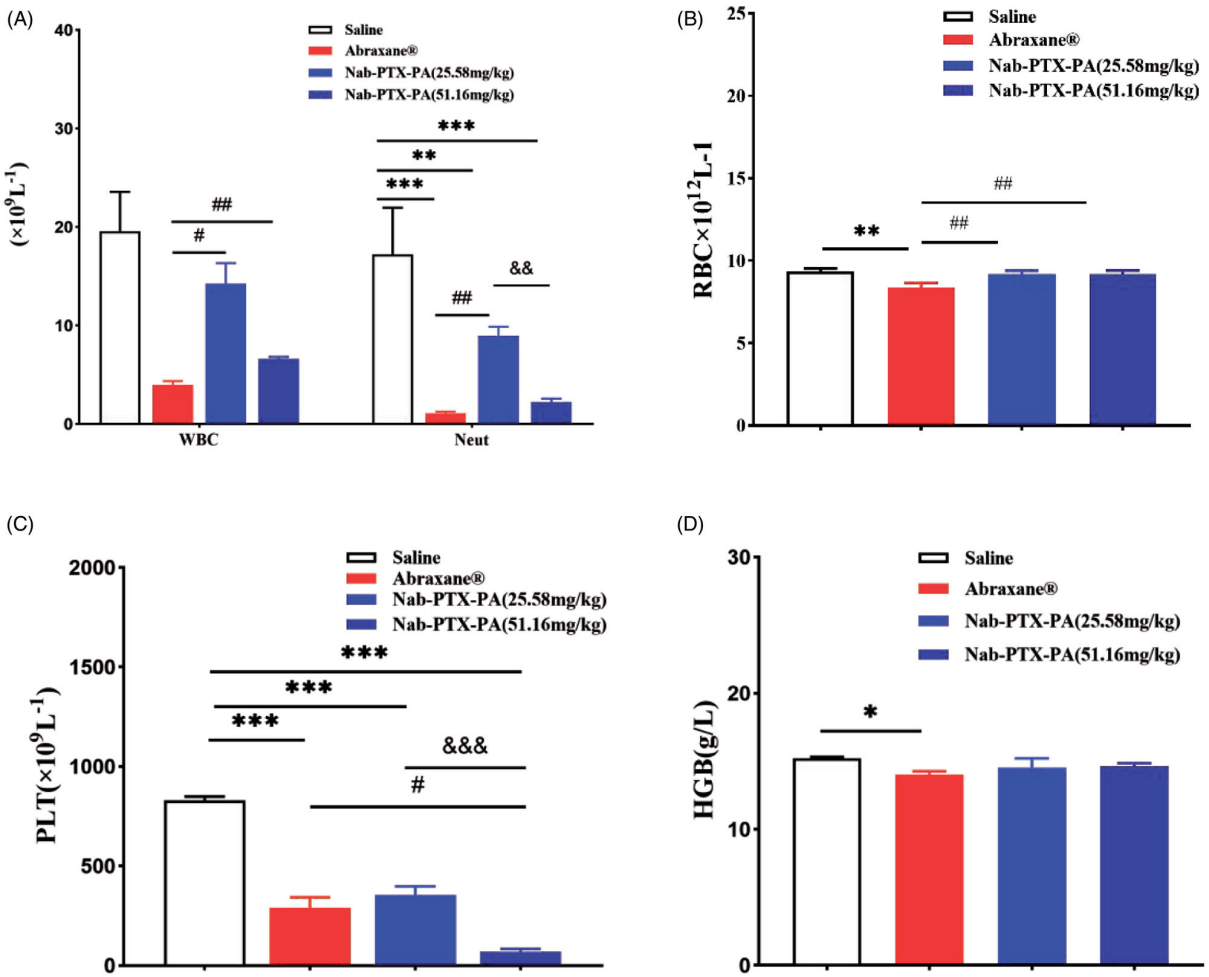
Blood routine examination. (A) WBC, Neut (×10^9^ L^−1^) (B) RBC (×10^12^ L^−1^) (C) PLT (×10^9^ L^−1^) (D) HGB (g/L).

**Table 6** shows single factor ANOVA analysis of the blood routine data using SPSS 22.0 software. Compared with NS group, the number of blood cells in the drug administration groups decreased in varying degrees. WBC (white blood cell) and RBC (red blood cell) were associated with myelosuppressive toxicity. Compared with paclitaxel albumin nanoparticles (20 mg/kg) group, the number of WBC and RBC were increased in which Nab-PTX-PA group (25.58 mg/kg) and Nab-PTX-PA (51.16 mg/kg) group, and statistically significant (*p* < .05). Neutropenia was a common adverse reaction of paclitaxel albumin nanoparticles. Figure 11 shows that the Neut of 25.58 mg/kg Nab-PTX-PA group was significantly improved as compared with 20 mg/kg paclitaxel albumin nanoparticles group.

**Table 6.** Comparison of blood parameters indicating the anti-tumor effect of Nab-PTX-PA in mouse 4T1 cells ($\bar{x}\pm \text{s},$ *n* = 6).

Group	WBC	RBC	PLT	Neut#	HGB
	(×10^9^L^-1^)	(×10^12^L^-1^)	(×10^9^L^-1^)	(×10^9^L^-1^)	(g/L)
Saline	19.54 ± 4.04	9.34 ± 0.19	829.67 ± 19.86	17.22 ± 4.73	15.20 ± 0.10
Abraxane^®^	4.01 ± 0.35	8.38 ± 0.27**	290.33 ± 52.54***	1.09 ± 0.17***	14.00 ± 0.26*
Nab-PTX-PA (25.58 mg/kg)	14.26 ± 2.08^&^	9.18 ± 0.21^&&^	353.67 ± 44.41***	8.97 ± 0.90^**, &&^	14.53 ± 0.68
Nab-PTX-PA (51.16 mg/kg)	6.64 ± 0.17^&&^	9.17 ± 0.24^&&^	69.00 ± 14.18^***, &, ▲▲▲^	2.24 ± 0.33^***, ▲▲^	14.63 ± 0.21

Label a: Comparison with negative control (saline)：*p < .05, **p < .01, ***p < .001.

Label b: Comparison with Abraxane® group：^&^*p* < .05, ^&&^*p* < .01.

Label c: Comparison with Nab-PTX-PA (25.58 mg/kg) group：^▲▲^*p* < .01, ^▲▲▲^*p* < .001.

### 3.10. Blood biochemical test

**Figure 12.**
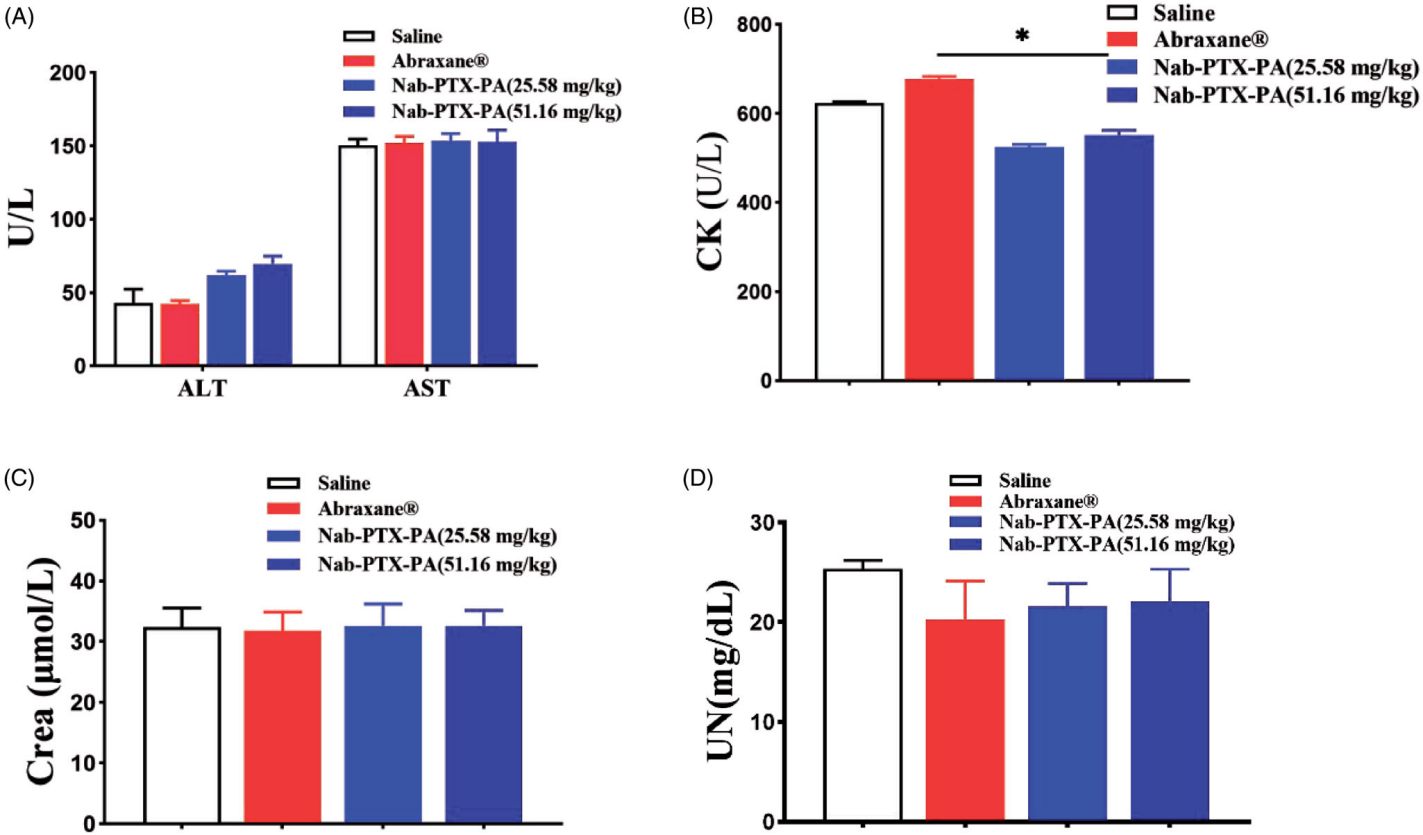
Blood biochemical tests. (A) ALT, AST (U/L), (B) CK (U/L), (C) Crea (μmol/L), (D) UN (mg/dL).

As Table 7 shows, Blood biochemical index test showed no significant difference in AST, ALT, UN and CR levels between the four groups, indicating that Nab-PTX-PA did not have significant adverse effects on liver and kidney functions. Bone marrow suppression toxicity and cardiotoxicity are important indexes for the application of PTX-based preparations. The results demonstrated no significant difference in CK levels between Nab-PTX-PA and NS groups. While CK level was elevated significantly in paclitaxel albumin nanoparticles group (Figure 12), suggesting that Nab-PTX-PA did not induce cardiotoxicity at the designed doses. Hemocompatibility, histocompatibility and the enhanced antitumor effect of Nab-PTX-PA could be attributed to the palmitic acid modification and targeted sustained.

**Table 7.** Comparison of blood parameters indicating the anti-tumor effect of Nab-PTX-PA in mouse 4T1 cells (±s, *n* = 6).

Group	ALT	AST	UN	Crea	CK
	(U/L)	(U/L)	(mg/dL)	(μmol/L)	(U/L)
Saline	42.8 ± 10.98	150.5 ± 4.27	25.34 ± 1.42	32.4 ± 5.17	623.4 ± 32.50
Abraxane	42.2 ± 8.23	152.1 ± 0.87	20.23 ± 2.11	31.8 ± 1.29	676.5 ± 15.32
Nab-PTX-PA (25.58 mg/kg)	61.7 ± 0.87	153.4 ± 1.87	21.59 ± 0.76	32.6 ± 2.88	524.6 ± 20.43
Nab-PTX-PA (51.16 mg/kg)	69.6 ± 1.55	152.9 ± 0.64	22.12 ± 0.45	32.1 ± 3.87	550.7 ± 15.55

## 4. Discussion

In this experiment, Nab-PTX-PA was prepared by using chloroform and anhydrous ethanol as the solvent, emulsified and homogenized under high-pressure homogenization without using the surfactant. The operation process was simple, and the liposolubility of PTX-PA was stronger than PTX. During the preparation process, albumin was not easy denatured at low temperature, and finally the organic solvent was removed by ultrafiltration. PTX-PA was more stable in an amorphous state and not easy to leak during storage, thus improving the stability of the product. The prepared Nab-PTX-PA was spherical and evenly distributed, with a particle size of 87.63 ± 1.15 nm (*n* = 3), a single peak, PDI = 0.185 ± 0.009 (*n* = 3), and Zeta potential of −11.70 ± 0.61 mV (*n* = 3). The result of the experiment with Nab-PTX-PA in vitro release and the degradation of PTX-PA into PTX showed that Nab-PTX-PA could release PTX-PA from plasma and transform into active mother drug PTX under the action of enzyme ester in vivo, which is consistent with the expectation of the experiment. After conversion to the mother drug, the activity of 2-OH was restored, which provided the possibility to achieve the anti-tumor effect. PTX was Modified to PTX-PA by one step esterification, the more fat-soluble, the stronger it bound within hydrophobic pockets of albumin, and the steric hindrance of the ester group increases, which made hydrolysis difficult, and the main purpose of preparing Nab-PTX-PA was to slow down the release.

The ability of drug uptake by cells is an important index to reflect the efficacy. The results of cell uptake experiments showed that Nab-PTX-PA could be ingested by 4T1 tumor cells and had a potential anti-tumor effect. The result of cell proliferation toxicity in vitro showed that the Nab-PTX-PA had concentration-dependent toxicity, and the cell proliferation toxicity was significantly lower than that of the commercially available paclitaxel albumin nanoparticles. On the basis of in vitro experiments, we performed a tissue distribution experiment and a pharmacodynamics study to investigate bone marrow suppression of the testing drugs. The result of the tissue distribution experiment showed that the concentration of PTX metabolized by Nab-PTX-PA increased slowly in the tumor site, which proved that Nab-PTX-PA could be continuously transformed into PTX to increase its accumulation in the tumor site. As a result, the anti-tumor potential was increased. After intravenous injection of paclitaxel albumin nanoparticles, the drug concentration in the liver and kidney was relatively high, and the drug concentration decreased rapidly in various tissues of the mice, and the same trend was also observed in the tumor site, indicating that the time of the anti-tumor action of paclitaxel albumin nanoparticles was shortened after it entered the body. After injection of Nab-PTX-PA into the tail vein, the concentration of PTX-PA distribution was increased in all tissues except the tumor site, indicating a wide range of PTX-PA distribution, but the prodrug itself was not active. So it was speculated that PTX-PA had relatively small damage to the normal organs, and the concentration of metabolized PTX increased gradually in various tissues, with the liver and lung predominating, which is consistent with the general characteristics of tissue distribution of NPs and has proved to have no special toxicity. At the tumor site of the tumor-bearing mice, the metabolic PTX concentration increased gradually, which is more beneficial to the anti-tumor effect in the tumor site for a long time. The results of pharmacodynamic study in vivo showed no significant difference in the anti-tumor effect between Nab-PTX-PA (25.58 mg/kg) and Abraxane (20 mg/kg) groups, and the toxicity of bone marrow suppression was decreased in both groups. In addition, 51.16 mg/kg Nab-PTX-PA and Abraxane (20 mg/kg) had similar myelosuppression toxicity, but the anti-tumor effect of the former was significantly enhanced.

As the volume ratio of organic phase to aqueous phase decreased from 1:9 to 1:22, the particle size also decreased to about 85 nm. Under the action of the emulsifier, the organic phase was dispersed into fine emulsion droplets. The smaller the concentration of the emulsion droplets in the dispersed medium, the smaller the relative viscosity will be, and the more uniform the emulsion dispersion, the smaller the chance of agglomeration between emulsions will be. As a result, a relatively stable dispersion system can be formed. However, when the volume ratio decreases to a certain extent, the particle size of NPs would not change much. It is proved that the mass concentration of the carrier material affects the deposition rate of NPs in the process of ball formation. With the increase of the polymer mass concentration, the particle size increases accordingly. After the particle size decreases, it is not easy to be swallowed by the reticular endothelial system as a foreign body, the half-life is prolonged, the rate of clearance from plasma slows down, and the targeting effect is enhanced.

Compared with Abraxane (drug loading is approximately 10%), the drug loading of Nab-PTX-PA (18.30%, turning into the drug loading of paclitaxel is 14.31%) are relatively high. It is speculated that the solubility of the PTX itself is poor. After palmitic acid modification, the liposolubility is greatly improved, the log*p* value is in the suitable range of 0–3, which improves the properties of the preparation and makes the drug easy to be absorbed. Nab^TM^ technology uses the cavitation under high shear force to open the sulfhydryl or disulfide bond between albumin, and then cross-links between albumin to form new disulfide bonds, thus preparing the NPs. Because of the low temperature in the preparation process, albumin is not easy to denature. In addition, albumin contains a hydrophobic domain and has a variety of hydrophobic drug binding sites, which can better encapsulate hydrophobic drugs.

Myelosuppressive toxicity is the main side effect of paclitaxel albumin nanoparticles, which can cause coagulation dysfunction and increase the risk of infection or even secondary leukemia, thus limiting its clinical use. Blood routine examination of the tumor-bearing mice showed that Nab-PTX-PA (25.58 mg/kg) could significantly reduce the toxicity of bone marrow suppression. It was speculated that when PTX was prepared into a PTX-PA prodrug, the toxicity of bone marrow suppression would be decreased according to the characteristics of the prodrug. Compared with the commercially available paclitaxel albumin nanoparticles(20 mg/kg), Nab-PTX-PA (25.58 mg/kg) showed no significant difference in antitumor efficacy but had less myelosuppressive toxicity. Compared with paclitaxel albumin nanoparticles (20 mg/kg), Nab-PTX-PA (51.16 mg/kg) had similar myelosuppressive toxicity but its anti-tumor effect was increased significantly. Presumably, Nab-PTX-PA at the same concentration is less toxic than paclitaxel albumin nanoparticles, which provides a new research idea for the development of PTX preparations.

Nab-PTX-PA (25.58 mg/kg) showed the same anti-tumor effect as the commercial drug Abraxane (20 mg/kg) in the tumor-bearing mice, and the anti-tumor effect of 51.16 mg/kg Nab-PTX-PA was better than that of Abraxane (20 mg/kg). It is speculated that PTX-PA has high-fat solubility, binds more closely to albumin, and slowly forms a drug-protein complex in blood. More drugs enter tumor cells through active targeting mediated by gp60-cellar protein-SPARC. Palmitic acid is a fatty acid that can be absorbed as energy by cancer cells, so the more the drug accumulates in the tumor site, the stronger the ability to inhibit tumor growth. Albumin is an endogenous substance with no immunogenicity and toxicity. Modification of PTX with palmitic acid not only reduced the adverse effects of the drug but increased tolerance of the experimental mice to the drug. The particle size of Nab-PTX-PA is about 86 nm, and, therefore, the drug can penetrate and remain in the tumor site more easily through the EPR effect of the tumor, and exert a better anti-tumor effect. In addition, there may be a metabolic balance between the degradation of Nab-PTX-PA to PTX and the consumption of PTX in mice in vivo. In the tumor site, there may be a large number of metabolic esterases with different degrees of activity. After palmitic acid modification, Nab-PTX-PA is doubly targeted, and under the action of metabolic esterase, the directional release of PTX at the tumor site makes the anti-tumor effect of Nab-PTX-PA more effective. On the basis of the above research, our team will continue to study the tissue distribution, pharmacokinetics and different metabolic esterase activities of Nab-PTX-PA in vivo and try to explain the results of pharmacodynamic evaluation of Nab-PTX-PA in animals at s deeper level.

## 5. Conclusion

In the present study, given the relationship between HSA, fatty acids, and the tumor microenvironment, this is a potential strategy for drug delivery to cancers that have increased demands for HSA and fatty acids. we constructed a drug delivery system by combining the prodrug technology with albumin NPs to overcome the PTX toxicity, short half-life, fast metabolism and poor water solubility, knowing that it is difficult to prepare albumin NPs. Nab-PTX-PA that we prepared in this study has the advantages of high drug loading, good stability and injectability. The retention time of Nab-PTX-PA at the injection site is more than 40 days. Nab-PTX-PA showed a significant inhibitory effect on tumor growth and metastasis in 4T1 tumor-bearing mice in vivo with low systemic cytotoxicity. In addition, Nab-PTX-PA prepared by the Nab^TM^ technology has no inert carrier or drug adjuvant with an organic solvent. Compared with the commercially available PTX preparations, Nab-PTX-PA has better physical stability and drug loading. According to the EPR mechanism, Nab-PTX-PA can effectively deliver PTX to the tumor environment. In addition, Nab-PTX-PA improved the pharmacokinetic parameters of PTX and prolonged the half-life of PTX. Compared with other preparations, the dual targeting of Nab-PTX-PA prodrug and albumin also improved the anti-tumor activity and survival rate of animals. To sum up, this Nab-PTX-PA preparation is safe and effective, showing broad application prospects in tumor targeted therapy, and may promote the further development of PTX in practical application.
